# Spatial–Temporal Pattern and Convergence Characteristics of Provincial Urban Land Use Efficiency under Environmental Constraints in China

**DOI:** 10.3390/ijerph191710729

**Published:** 2022-08-29

**Authors:** Rongtian Zhang, Jianfei Lu

**Affiliations:** Institute of Rural Revitalization Strategy, Yangzhou University, Yangzhou 225009, China

**Keywords:** urban land use efficiency, spatial–temporal pattern, convergence, environmental constraints, provincial scale

## Abstract

Revealing the spatial–temporal pattern and convergence characteristics of urban land use efficiency has important guiding significance for adjusting and optimizing the regional urban land use structure. Taking the provincial units in China as the research object, the urban land use efficiency evaluation system considering the unexpected output was constructed, and the slack-based measure (SBA) model was used to quantitatively measure the provincial urban land use efficiency from 2000 to 2020. The exploratory spatial data analysis (ESDA) model and spatial convergence index were combined to reveal the spatial–temporal pattern and convergence characteristics of provincial urban land use efficiency. The results showed that the provincial urban land use efficiency has been continuously improving, with regional differences as shown in eastern region > northeast region > central region > western region. Moran’s I of provincial urban land use efficiency was greater than 0, there was a positive spatial correlation, and the clustering feature became increasingly significant. The spatial form of LISA was characterized by “small agglomeration and large dispersion”; the H(High)-H(High) type was clustered in the Yangtze River Delta and Beijing–Tianjin–Hebei, while the L(Low)-L(Low) type was clustered in Xizang, Xinjiang and Qinghai. There was a σ convergence in provincial urban land use efficiency, and there was significant absolute β convergence and conditional β convergence of provincial urban land use efficiency. The results showed that the differences in provincial urban land use efficiency were shrinking, showing a “catch-up effect”, and converging to their respective stable states over time. Based on the analysis of the spatial–temporal pattern and convergence characteristics of provincial urban land use efficiency in China, we could provide a direction for the optimization of the urban land use structure and efficiency improvement in China, in order to narrow the differences in urban land use efficiency in China’s four major regions.

## 1. Introduction

The land resource is the scarcest resource. As a spatial carrier of the urban economy, society and environment, the improvement of its utilization efficiency is an important goal of land reform, and also an inherent requirement for the transformation of the economic development mode and the construction of ecological civilization under the new normal [1]. Since the reform and opening up, along with the rapid advancement of urbanization and industrialization in China, the amount of urban land has shown a continuous surge. At the same time, the extensive urban land use pattern also leads to a series of problems, such as an unreasonable land structure, disordered land distribution and low land use efficiency [2]. In the process of rapid urbanization, the excessive consumption of resources and environmental pollution are increasing, and China’s urbanization development is approaching the constraint boundary of resources and environmental conditions. The scarcity and extensive utilization of land resources have become important factors restricting the high-quality development of cities in China. Therefore, how to effectively improve the efficiency of urban land resource utilization has become a key problem to be solved in the process of China’s new urbanization construction.

Urban land use efficiency belongs to the category of intensive land use, which is aimed at extensive land use. The concept originated from the research ideas of land rent, such as Turgot and Ricardo in classical economics. In the early 19th century, Thunnen proposed the rational distribution and layout of agricultural land in the study of the relationship between location land rent and urban distance, which became a milestone in the study of urban land use [3]. Subsequently, Weber’s industrial location theory, Taylor’s central place theory and other classical theories enriched the research on the spatial structure of urban land use [4]. In the 1920s, the ecological school rose and constructed three classical models of urban land use structure, which laid an important foundation for the study of urban land use efficiency [5]. At the same time, the continuous development of social science theory and the introduction of spatial economics, behavior analysis and other research methods gradually formed the economic location school, social behavior school and political economy school and other research theoretical systems [6]. Since the 1990s, urban land use has become more and more comprehensive, and research has moved towards the direction of integration. Therefore, the theory of sustainable urban land use has arisen at a historic moment. It not only focuses on the efficiency of land use structure in urban development, but also focuses on the direction of urban land use in the future, seeking fair, reasonable and efficient use of urban land. In general, the research focus has shifted from the early ecological school to the spatial succession model to the exploration of the decision-making process and dynamic mechanism of urban land development, and from advocating the free operation of the land market to the smart management of land development.

Urban land use efficiency is a comprehensive concept; from the perspective of comprehensive utilization of resources, it includes the economic benefits, social benefits and environmental benefits of land [7]; from the perspective of factor input, it measures the utilization efficiency of land factors in urban economic growth [8]. On the basis of previous studies, we believes that urban land use efficiency is the process of putting production factors such as population and capital into land resources, and obtaining the optimal land economic value output, social value output and environmental value output through a series of social and economic activities, while causing the minimum environmental pollution. At present, the study of urban land use efficiency has become one of the hot topics in economics, management and geography. Combing through relevant research results, research on urban land use efficiency has mainly focused on connotation interpretation [9,10], regional differences [11,12,13], influencing factors [14,15], driving mechanisms [16,17] and optimization suggestions [18,19,20]. Some scholars have also begun to pay attention to the differences and convergence of land use efficiency in cities of different sizes [21,22]. In terms of research methods, the measurement methods of urban land use efficiency mainly fall into two categories, non-parametric methods and parametric methods, corresponding to two classical models, data envelope analysis (DEA) [23,24] and stochastic frontier analysis (SFA) [25,26]. The DEA model does not need to set specific function forms; the SFA model needs to set the function form of production function [27]. In addition, the GIS analysis model [28], ESDA [29] and other spatial metrology methods have also been applied in the evaluation of land resource utilization efficiency. In terms of research objects, the urban development zone [30], single cities [31] and urban agglomeration [32] were mainly involved in different scales of spatial regions, and the research on urban land use efficiency presents a trend of multi-scale development. It could be found that there are still some shortcomings in the current research: first, previous studies on the evaluation of urban land use efficiency paid more attention to the expected output of economic and social benefits, but ignored the unexpected output of ecological environment pollution in the process of urban land use [33,34,35]. Second, most of the analysis results were based on the phenomenon description and mechanism explanation from the perspective of economy or management, and the time series expression of the geographic spatial form of urban land resource use efficiency was relatively weak [36,37].

**Figure 1:**
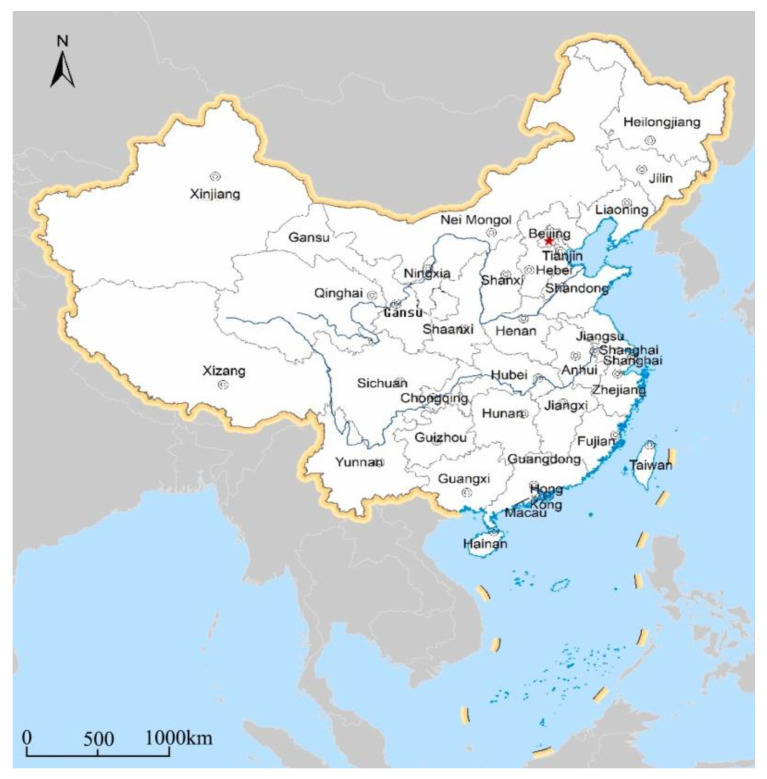
Study area.

Therefore, this study took the provincial units in China as the research object (Figure 1); the evaluation index system of urban land use efficiency considering environmental pollution and other undesirable outputs was constructed, and we comprehensively applied a variety of methods to study the spatial–temporal pattern and convergence characteristics of urban land use efficiency at the provincial level in China. The structure of the manuscript mainly includes three parts, as follows.

- How to measure urban land use efficiency considering unexpected output. In this part, from the perspective of input–output, the evaluation index system of urban land use efficiency considering the effect of resources and environment was constructed, and the SBM model was used to measure the level of urban land use efficiency in China since 2000.
- How to reveal the spatial–temporal differences in provincial urban land use efficiency in China. In this part, based on the ESDA model, the spatial–temporal pattern of urban land use efficiency at the provincial level was analyzed from the perspectives of global autocorrelation and local autocorrelation since 2000.
- How to reveal the convergence characteristics of provincial urban land use efficiency in China. In this part, we used the σ convergence, absolute β convergence and conditional β convergence index to empirically analyze the spatial convergence characteristics of provincial urban land use efficiency since 2000.

## 2. Materials and Methods

### 2.1. Index System

The construction of an evaluation index system was the basis for the empirical analysis of urban land use efficiency. As for the evaluation index of urban land use efficiency, there has not been a relatively unified standard. In general, the land use system was divided into two subsystems, input and output, and urban land use efficiency could be measured by input and output [38,39,40]. On this basis, from the research perspective of input–output, this study constructed an evaluation index system of urban land use efficiency considering undesired output (Table 1). ① The input dimension mainly reflected the human resources, capital and land input in the process of urban land use. In this part, urban population (*X*_1_) was mainly selected to reflect the human input of urban land use; annual capital stock (*X*_2_) and foreign direct investment (*X*_3_) were selected to reflect the capital input of urban land use; urban land area (*X*_4_) was selected to reflect the land input of urban land use. ② The output dimension mainly reflected the expected outputs, such as economic benefits and social benefits, in the process of urban land use, as well as the unexpected outputs, such as ecological environment pollution. In this part, GDP (*X*_5_) was selected to reflect the economic output of urban land use; the total retail sales of consumer goods (*X*_6_) was selected to reflect the social output of urban land use; three indicators of urban industrial waste water discharge (*X*_7_), urban industrial waste gas discharge (*X*_8_) and urban industrial solid waste production (*X*_9_) were selected to reflect the unexpected output of urban land use.

### 2.2. Data Collection

The analysis of data in this paper is mainly included two parts: statistical data and spatial data. ① Statistical data. The analysis indexes in the evaluation index system mainly came from the China Urban Statistical Yearbook (2001–2019), China Regional Economic Statistical Yearbook (2001–2019) and the statistical yearbook of China’s 31 provincial units (2001–2019). ② Spatial data. The spatial scale of the spatial–temporal feature analysis in this study was provincial units, including 31 provincial units in China (Hong Kong, Macao and Taiwan were not included due to limited data). The provincial administrative region boundary data came from the atlas of China, which was obtained by high-precision registration and tracking vectorization in ArcGIS 10.2 software (Environmental Systems Research Institute, Redlands, CA, USA).

### 2.3. Research Methods

#### 2.3.1. SBM Model

The slack-based measure (SBA) model is an analytical method to evaluate the relative effectiveness of input–output by setting a number of input and output indicators and using linear programming and comparable decision-making units. This method effectively overcomes the problem that the radial DEA model does not consider the slack of input and output, and considers the problem of undesirable output contained in the process of urban land use [41,42]. In view of this, the study used the SBM model to measure the value of provincial urban land use efficiency considering unexpected output. The SBM theoretical model is as follows:

(1) $$\rho =\min\frac{1-\frac{1}{N}\sum_{n=1}^{N}S_{n}^{x}/x_{k^{\prime }n}^{t^{\prime }}}{1+\frac{1}{M+I}\left(\sum_{m=1}^{M}s_{m}^{y}/y_{k^{\prime }m}^{t^{\prime }}+\sum_{i=1}^{I}s_{i}^{b}/b_{k^{\prime }i}^{t^{\prime }}\right)}$$

where ρ is the value of urban land use efficiency; *n*, *m* and *i* are the number of inputs, expected outputs and unexpected outputs, respectively; $\left(s_{n}^{x},s_{m}^{y},s_{i}^{b}\right)$ represents the relaxation vector of input and output; $\left(x_{k^{\prime }n}^{t^{\prime }},y_{k^{\prime }m}^{t^{\prime }},b_{k^{\prime }i}^{t^{\prime }}\right)$ is the input–output value of region *k*’ in period *t*’; $z_{k}^{t}$ represents the weight of the decision-making unit; for ρ, the value range is [0, 1].

#### 2.3.2. ESDA Model

The exploratory spatial data analysis (ESDA) model describes and visualizes the spatial distribution pattern of geographical phenomena, and discovers the spatial clustering characteristics and analyzes the mechanism. The specific indicators of the model include global Moran’s I and the LISA index [43,44], and the theoretical formula is as follows.

① Global Moran’s I index

(2) $$I(d)=\frac{\sum_{i=1}^{n}\sum_{j=1}^{n}\left(X_{i}-\bar{X}\right)\left(X_{j}-\bar{X}\right)}{S^{2}\sum_{i=1}^{n}\sum_{j=1}^{n}W_{ij}}$$

(3) $$S^{2}=\sum_{i=1}^{n}{\left(X_{i}-\bar{X}\right)}^{2}/n$$

where $X_{i}$ is the observed value of the region i; $X_{j}$ is the observed value of the region j; $W_{ij}$ is the spatial weight matrix, and the spatial adjacency is 1. I(d) > 0 is a positive spatial correlation; it indicates that urban land use efficiency is high (low), and there is significant agglomeration in space.

② LISA index

(4) $$I_{i}={Z^{\prime }}_{i}\sum_{j=1}^{n}W_{ij}{Z^{\prime }}_{j}$$

where $Z_{i}^{’}$ and $Z_{j}^{’}$ are the standardization of observed values on regions i and j. Here, $I_{i}>0$ indicates that urban land use efficiency has a small difference from surrounding areas. Meanwhile, $I_{i}<0$ indicates that the urban land use efficiency is significantly different from the surrounding area.

#### 2.3.3. Convergence Index

Convergence of urban land use efficiency refers to the process in which there is a negative correlation between the initial static index and growth rate for different evaluation units, so that the differences in the initial static index of each evaluation unit gradually disappear. There are three types of convergence indices: σ convergence, absolute β convergence and conditional β convergence [45].

① σ convergence

σ convergence is mainly used to measure the change in urban land use efficiency difference with time. If the σ value decreases over time, i.e., the dispersion degree of urban land use efficiency in the province gradually decreases, it is called σ convergence. The σ convergence index model can be expressed as [46]

(5) $$\sigma_{t}=\sqrt{\frac{1}{n-1}\overset{n}{\underset{i=1}{\sum }}{\left(E_{i,t}-E_{t}\right)}^{2}}/E_{t}$$

where $E_{i,t}$ is the urban land use efficiency value of the *i*th province at *t*; when $E_{t}$ is *t*, the mean value of urban land use efficiency in *n* provinces is obtained. If ${\;\sigma }_{t}>\sigma_{t+1}$, there is σ convergence, i.e., the difference between provincial urban land use efficiency decreases gradually over time.

② Absolute β convergence

Absolute β convergence means that the urban land use efficiency of different provinces will eventually converge to the same equilibrium level. Absolute β convergence mainly measures the negative correlation between variables and the initial level of variables to judge whether there is a “catch-up effect”. The theoretical model is as follows [47]:

(6) $$\frac{\left[\ln\left(E_{i,t}\right)-\ln\left(E_{i,0}\right)\right]}{T}=\alpha +\beta \ln\left(E_{i,0}\right)+\varepsilon$$

where $\;E_{i,0}$ is the urban land use efficiency of the *i*th province in the early stage of a certain period; $E_{i,t}$ is the urban land use efficiency of the *i*th province at the end of a certain period; α is a constant term; β is the estimated coefficient; ε is the random error term. If β < 0 and it passes the significance test, absolute β convergence exists.

③ Conditional β convergence

Conditional β convergence means that different provincial units have different equilibrium growth paths and converge to different steady states. The purpose is to judge whether the measurement of provincial urban land use efficiency converges to its own steady state. The theoretical model is as follows [48]:

(7) $$\ln\left(E_{i,t}\right)-\ln\left(E_{i,t-1}\right)=\alpha +\beta \ln\left(E_{i,t-1}\right)+\varepsilon$$

where $E_{i,t-1}$ and $E_{i,t}$ are the urban land use efficiency values of the *i*th provincial unit at *t* − 1 and *t*, respectively; α is a constant term; β is the convergence coefficient; ε is the random error term. If β < 0 and it passes the significance test, the conditional β convergence exists.

## 3. Results

### 3.1. Temporal Variation Characteristics of Urban Land Use Efficiency

Based on the original input–output data of 31 provincial units in China, the SBM mathematical model was used to calculate the level of urban land use efficiency at the provincial level in China from 2000 to 2020 (Table 2). The following observations can be derived from Table 2. ① General characteristics. Considering the unexpected output and not considering the unexpected output, the level of urban land use efficiency in China’s provinces showed a certain improvement trend. Considering the unexpected output, China’s provincial urban land use efficiency fluctuated and increased in the range of [0.6128, 0.6491] from 2000 to 2020, and the urban land use efficiency has increased by 5.91% in the past 20 years. However, without considering the unexpected output, the value of China’s provincial urban land use efficiency changed in the range of [0.6193, 0.6556] during the study period. In contrast, the level of urban land use efficiency considering unexpected output was relatively more reasonable and scientific than that without unexpected output. The study mainly analyzed the difference in the urban land use efficiency level between the two different situations by comparing and analyzing the two basic situations of considering the unexpected output and not considering the unexpected output, so it can be seen that the urban land use efficiency considering the unexpected output was more scientific and reasonable.

**Figure 2:**
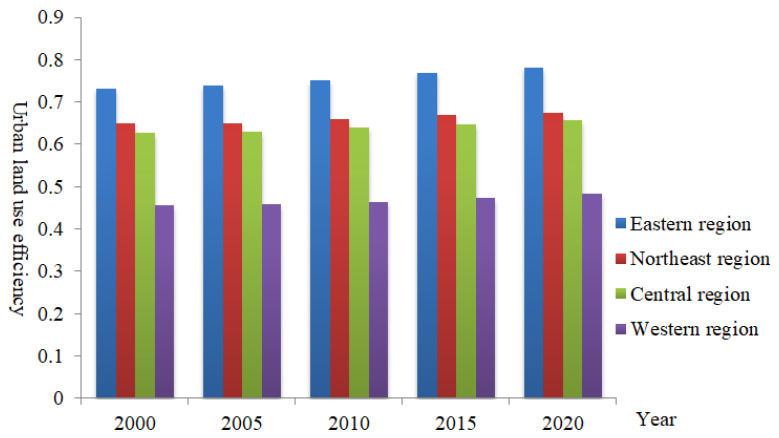
Regional differences in provincial urban land use efficiency from 2000 to 2020.

② Regional differences. From 2000 to 2020, China’s provincial urban land use efficiency showed significant regional differences. Specifically, the average level of urban land use efficiency in the eastern region changed in the range of [0.7326, 0.7809], the average level of urban land use efficiency in the northeast region fluctuated in the range of [0.6487, 0.6751], and the average level of urban land use efficiency in the central region changed in the range of [0.6276, 0.6570]. Meanwhile, the average level of urban land use efficiency in the western region fluctuated in the range of [0.4559, 0.4837]. Overall, the level of urban land use efficiency in China’s provinces showed the regional differentiation law of “high in the east and low in the west” during the whole study period (Figure 2).

### 3.2. Spatial Pattern Evolution of Urban Land Use Efficiency

#### 3.2.1. Global Spatial Pattern

Based on GeaDA095 software (Environmental Systems Research Institute, Redlands, CA, USA), the global Moran’s I value of China’s provincial urban land use efficiency was calculated from 2000 to 2020 (Table 3). At the significance test level of 0.1%, the global Moran’s I values of provincial urban land use efficiency were positive. This indicated that the provincial urban land use efficiency showed positive autocorrelation during the study period, i.e., the level of urban land use efficiency of geographically adjacent provincial units had relative spatial agglomeration. Meanwhile, it could be found that the global Moran’s I value of China’s provincial urban land use efficiency increased from 0.3558 in 2000 to 0.4025 in 2020, i.e., the global Moran’s I value increased by 13.13%, showing a continuous upward evolution trend. This showed that with the passage of time, the positive correlation characteristics of China’s provincial urban land use efficiency were gradually strengthened, and the spatial agglomeration became more and more significant.

#### 3.2.2. Local Spatial Differentiation

**Figure 3:**
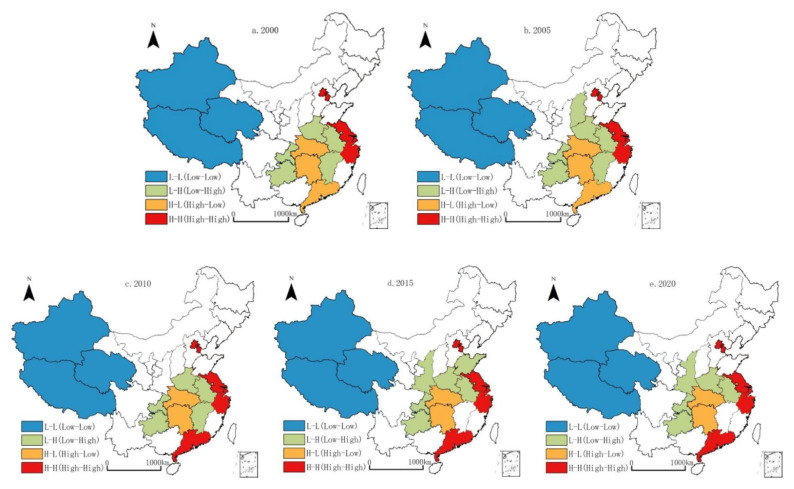
LISA evolution of provincial urban land use efficiency from 2000 to 2020.

Global autocorrelation only analyzed the overall characteristics of the spatial distribution of urban land use efficiency at the provincial level, and it was difficult to reveal its internal differentiation law [49]. Therefore, in order to further explore the spatial correlation degree of provincial urban land use efficiency, the study used the local autocorrelation index and drew a LISA cluster map of the provincial urban land use efficiency from 2000 to 2020 based on ArcGIS10.2 software (Figure 3).

H-H type (the urban land use efficiency of the province itself and adjacent provinces was high). In 2000, the H-H type of provincial urban land use efficiency was mainly distributed in Shanghai, Jiangsu and Zhejiang, as well as Beijing and Tianjin. By 2020, Guangdong evolved into the H-H type. On the whole, provincial urban land use efficiency of the H-H type was mainly concentrated in the Yangtze River Delta, Pearl River Delta and Beijing–Tianjin–Hebei. This type was associated with the regions with rapid economic development in Eastern China; urban industrial transformation and land system reform were at the forefront, which had a continuous promotional effect on the improvement of urban land resource efficiency.

H-L type (the urban land use efficiency of the province itself was high while that of the neighboring provinces was low). In 2000, the H-L type of provincial urban land use efficiency was mainly distributed in Hunan, Hubei and Guangdong. By 2010, Guangdong province evolved into the H-H type; after this, the H-L pattern did not change. This type of provincial unit urbanization was also relatively developed, and the industrial structure was constantly transformed and upgraded, which also helped to improve the efficiency of urban land resource use.

L-H type (the urban land use efficiency of the province itself was low while that of the neighboring provinces was high). During the study period, the L-H type of provincial land use efficiency was mainly concentrated in Anhui, Henan, Shanxi, Chongqing, Guizhou, Shaanxi, Jiangxi and Shandong; only Shanxi, Shaanxi and Jiangxi have been replaced. This type of provincial unit urbanization was in a relatively slow development stage, the urban industrial structure was relatively backward, and the land resource utilization mode was relatively extensive, resulting in the relatively low efficiency of urban land resource utilization in 2000.

L-L type (the urban land use efficiency of the province itself and adjacent provinces was low). From 2000 to 2020, the L-L-type space was mainly concentrated in Xinjiang, Xizang and Qinghai, and the L-L type pattern was not replaced. This type of provincial unit had complex landforms, relatively slow urbanization and industrialization, a lack of land resources and an extensive utilization mode, forming a stable L-L collapse area of provincial urban land use efficiency in China during the study period. 

In general, during the study period, the spatial pattern of the local LISA of China’s provincial urban land resource use efficiency was characterized by “small concentration and large dispersion”, and the differentiation pattern was basically stable. The LISA index clearly revealed the spatial differentiation characteristics of provincial urban land use efficiency in China from 2000.

### 3.3. Convergence Characteristics of Urban Land Use Efficiency

The spatial–temporal characteristics of provincial urban land use efficiency in China have been revealed in this study, but what was the convergence trend of provincial urban land use efficiency evolution? Convergence analysis can explore the convergence and divergence of provincial urban land use efficiency in China in the future. If the provincial urban land use efficiency has the characteristics of convergence, it indicates that the gap in provincial urban land use efficiency in China will gradually converge on a future time axis; otherwise, it will diverge [50,51]. In view of this, the study used the σ convergence, absolute β convergence and conditional β convergence index to empirically analyze the spatial convergence characteristics of provincial urban land use efficiency in China from 2000 to 2020.

#### 3.3.1. σ Convergence Test

**Figure 4:**
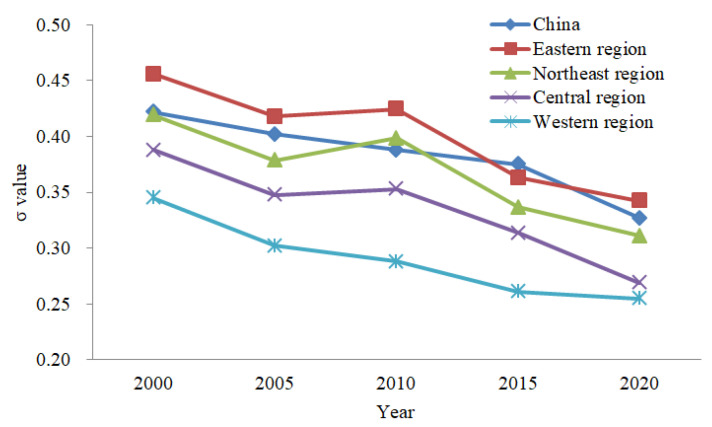
σ convergence trend of provincial urban land use efficiency in China and the four regions.

According to the σ convergence formula, the σ values of provincial urban land use efficiency in China and the eastern, central, western and northeastern regions from 2000 to 2020 were calculated (Figure 4). We made the following observations. ① On the whole, the σ value decreased from 0.4221 in 2000 to 0.3883 in 2010, and then to 0.3276 in 2020. It could be found that the σ value of provincial urban land use efficiency decreased during the study period, showing obvious σ convergence, indicating that the difference in urban land use efficiency at the provincial level in China will decrease over time. ② Regarding the sub-regions, from 2000 to 2020, σ value of provincial urban land use efficiency in the eastern, central, western and northeast regions fluctuated to different degrees, but all showed a downward trend, with the characteristic of σ convergence. With the rapid development of urbanization, the convergence characteristics of urban land use efficiency in the eastern region continued to be significant. This indicated that the internal differences in provincial urban land use efficiency in the four regions have gradually narrowed since 2000.

#### 3.3.2. Absolute β Convergence Test

Firstly, the absolute β convergence model was tested by the Hausman test using Stata, which showed that the fixed-effect regression model was more suitable. Secondly, the panel data estimation results of the absolute β convergence of urban land use efficiency in China and the four regions were calculated with the formula (Table 4). It could be seen from Table 4 that the β value of provincial urban land use efficiency in China and the eastern, central, western and northeastern regions was significantly negative after passing the significance test at the 1% level, indicating that there was absolute β convergence of provincial urban land use efficiency in China and the four regions. The analysis showed that the difference in urban land use efficiency at the provincial scale will decrease with the passage of time. The provinces with low efficiency of urban land resource use will grow at a fast speed and are catching up with the provinces with high efficiency of land resource use, and there is a “catch-up effect”. However, there were some regional differences in the catch-up speed during the study period; the convergence rate from high to low was western region > central region > northeast region > eastern region. 

#### 3.3.3. Conditional β Convergence Test

The conditional β convergence model can be regarded as a robustness test of the absolute β convergence model, and some variables were added to further verify the absolute β convergence model [52]. According to the absolute β convergence test results, control variables were added into the absolute β convergence model to investigate whether conditional β convergence exists in China and the eastern, central, western and northeastern regions. In the study, five impact indicators were selected as control variables, including per capita GDP (*Y*_1_), proportion of output value of secondary and tertiary industries (*Y*_2_), urban fixed investment (*Y*_3_), proportion of research funds (*Y*_4_) and land marketization index (*Y*_5_). Through Hausman’s test, it was confirmed that the fixed-effect model was supported for the whole country as well as the eastern, central, western and northeastern regions. The conditional β convergence test of provincial urban land use efficiency in China and the eastern, central, western and northeast regions was conducted by using the conditional β convergence index formula (Table 5).

Through Table 5, it can be see that after including five control variables, for both China and the eastern, central, western and northeast regions, β coefficients were less than 0 and passed the test of significance of 1%. This showed that there were significant conditions for urban land use efficiency in China and the four major regions’ β convergence. This analysis showed that the steady-state level exists in China and the eastern, central, western and northeast regions. At the same time, compared with the absolute β convergence analysis results, the goodness of fit of the conditional β convergence model was improved in both China and the eastern, central, western and northeastern regions; it also showed that the convergence of provincial urban land use efficiency was more significant after the introduction of the control variables.

## 4. Discussion

Based on the σ convergence, absolute β convergence and conditional β convergence index, the study analyzed the regional differences and convergence characteristics of provincial urban land use efficiency in China, and revealed that there has been σ convergence in provincial urban land use efficiency since 2000, indicating that the regional differences in urban land use efficiency at the provincial level in China are gradually decreasing. The absolute β and conditional β convergence of provincial urban land use efficiency in the eastern, central, western and northeastern regions were observed, indicating that the provincial urban land use efficiency in the four regions is developing towards a stable trend, but the differences among the four regions were still significant. Resource endowment, government policy and regional development level were the main factors causing the spatial–temporal differences and convergence differences in provincial urban land use efficiency in China from 2000. This provides a policy reference for us to formulate improvements in urban land use efficiency and the optimal allocation of urban land at the provincial level in China. At the same time, the next step is to deepen the systematic research on the influencing mechanism of the convergence characteristics of provincial urban land use efficiency in China and the eastern, central, western and northeastern regions.

As an important part of the National New Urbanization Planning, improving the efficiency of land resource use in regional cities is also one of the key tasks for the promotion of new urbanization. Based on the analysis of the spatial–temporal pattern and convergence characteristics of provincial urban land use efficiency in China, the following differentiated countermeasures and suggestions are proposed [53,54,55,56]. ① In the eastern and northeastern regions, we should strictly control the scale of urban land, economize and make intensive use of urban land resources, optimize the spatial distribution of urban land, pay attention to exploiting the spatial potential of the urban interior and promote the connotative development of urban land. We will encourage and guide the development of urban land into areas such as unused land and poor-quality agricultural land, strictly control the construction of new towns of all kinds and gradually reduce urban industrial land. At the same time, we will continue to promote the transformation and upgrading of urban industries, give full play to the role of the land market mechanism and comprehensively improve the overall efficiency of land use in urban agglomerations in the Yangtze River Delta, the Beijing–Tianjin–Hebei region and the Pearl River Delta. ② In the central and western regions, we need to focus on improving the technological innovation of urban land use and optimizing the allocation of land production factors, rather than blindly pursuing the scale efficiency of urban land use, controlling the spatial expansion of the urban land use boundary, realizing the reasonable control of the total scale of regional construction land and constantly reducing the dependence on urban land resources. At the same time, the central and western regions, as the key areas of industrial transfer in the eastern region, should strictly enforce the access standards and thresholds of transfer industries, pay attention to the protection of the urban ecological environment in the central and western regions and improve the comprehensive economic, social and ecological benefits of regional urban land use.

## 5. Conclusions

Provincial urban land use efficiency in China has been continuously improving since 2000. The value of provincial urban land use efficiency considering unexpected output changed in the range of [0.6128, 0.6491], which was lower than that without considering unexpected output [0.6193, 0.6556]. During the study period, the level of urban land use efficiency at the provincial level in China showed a significant regional difference of “high in the east and low in the west”.

The spatial–temporal pattern of provincial urban land use efficiency in China has been evolving since 2000. On the global level, from 2000 to 2020, the positive correlation characteristics of provincial urban land use efficiency have gradually strengthened, and the spatial agglomeration has become increasingly significant. On the local level, the H-H type of urban land use efficiency was mainly distributed in the Yangtze River Delta, Pearl River Delta and Beijing–Tianjin–Hebei region, while the L-L type was mainly concentrated in Xizang, Xinjiang and Qinghai in Western China. The local LISA agglomeration pattern of provincial urban land use efficiency showed the feature of “small agglomeration and large dispersion”.

There was σ convergence of provincial land use efficiency, which indicated that the difference in land use efficiency at the provincial level was narrowing. Absolute β convergence and conditional β convergence were observed in the eastern, central, western and northeast regions during the study period, indicating that the differences in provincial urban land use efficiency in China and the four regions would converge to a relatively stable state over time. Through the judgment of the convergence trend of provincial land use efficiency, this study provides an important reference for the classification and improvement of urban land use efficiency on the provincial scale in China.

## Figures and Tables

**Table 1:** Evaluation index system of urban land use efficiency.

Type	Indicators	Connotation
Input	Urban population (*X*_1_)	Human input
	Annual capital stock (*X*_2_)	Capital input
	Foreign direct investment (*X*_3_)	Capital input
	Urban land area (*X*_4_)	Land input
Output	GDP (*X*_5_)	Economic output
	The total retail sales of consumer goods (*X*_6_)	Social output
	Urban industrial waste water discharge (*X*_7_)	Unexpected output
	Urban industrial waste gas discharge (*X*_8_)	Unexpected output
	Urban industrial solid waste production (*X*_9_)	Unexpected output

**Table 2:** Urban land use efficiency in China and four major regions from 2000 to 2020.

Provincial Units		Considering the Unexpected Output			Not Considering the Unexpected Output		
		2000	2010	2020	2000	2010	2020
Eastern region	Beijing	0.7843	0.8213	0.8562	0.8012	0.8356	0.8643
	Tianjin	0.7523	0.7802	0.8283	0.7678	0.7953	0.8291
	Hebei	0.7032	0.7218	0.7389	0.7102	0.7314	0.7426
	Shanghai	0.7903	0.8289	0.8604	0.7993	0.8367	0.8688
	Jiangsu	0.7712	0.7934	0.8238	0.7823	0.8102	0.8332
	Zhejiang	0.7562	0.7721	0.7923	0.7603	0.7832	0.8021
	Fujian	0.7234	0.7403	0.7663	0.7304	0.7553	0.7777
	Shandong	0.7109	0.7339	0.7589	0.7267	0.7443	0.7637
	Guangdong	0.7689	0.7889	0.8203	0.7732	0.8089	0.8331
	Guangxi	0.6523	0.6709	0.6916	0.6604	0.6799	0.6998
	Hainan	0.6777	0.6903	0.7038	0.6795	0.6993	0.7078
	Mean	0.7326	0.7551	0.7809	0.7416	0.7667	0.7883
Northeast region	Jilin	0.6347	0.6402	0.6549	0.6388	0.6478	0.6601
	Heilongjiang	0.6113	0.6236	0.6403	0.6178	0.6289	0.6466
	Liaoning	0.7003	0.7189	0.7301	0.7078	0.7203	0.7378
	Mean	0.6487	0.6609	0.6751	0.6548	0.665667	0.6815
Central region	Shanxi	0.6234	0.6389	0.6541	0.6287	0.6402	0.6578
	Nei Mongol	0.5789	0.5903	0.6034	0.5801	0.5994	0.6089
	Anhui	0.6511	0.6669	0.6889	0.6589	0.6703	0.6912
	Jiangxi	0.6023	0.6115	0.6301	0.6078	0.6203	0.6378
	Henan	0.6312	0.6444	0.6603	0.6345	0.6502	0.6669
	Hubei	0.6634	0.6713	0.6923	0.6667	0.6789	0.6967
	Hunan	0.6525	0.6688	0.6891	0.6604	0.6789	0.6993
	Mean	0.6276	0.6395	0.6570	0.6326	0.6461	0.6628
Western region	Chongqing	0.5234	0.5337	0.5501	0.5278	0.5412	0.5553
	Sichuan	0.5122	0.5267	0.5405	0.5178	0.5323	0.5531
	Guizhou	0.4883	0.4923	0.5018	0.4903	0.4967	0.5089
	Yunnan	0.4729	0.4888	0.4978	0.4788	0.4904	0.5003
	Xizang	0.3234	0.3345	0.3552	0.3289	0.3404	0.3587
	Shanxi	0.5668	0.5812	0.5978	0.5703	0.5883	0.5998
	Gansu	0.4013	0.4234	0.4421	0.4109	0.4311	0.4563
	Qinghai	0.3678	0.3802	0.3995	0.3702	0.3899	0.4018
	Ningxia	0.5461	0.5555	0.5745	0.5498	0.5578	0.5804
	Xinjiang	0.3567	0.3689	0.3775	0.3603	0.3714	0.3837
	Mean	0.4559	0.4685	0.4837	0.4605	0.4740	0.4898

**Table 3:** Global Moran’s I values of provincial urban land use efficiency from 2000 to 2020.

Year	Global Moran’s I	E (I)	Z (I)	P (I)
2000	0.3558	−0.1212	2.08	0.001
2005	0.3662	−0.1211	2.19	0.001
2010	0.3804	−0.1211	2.32	0.001
2015	0.3928	−0.1213	2.39	0.001
2020	0.4055	−0.1214	2.52	0.001

Note: E (I) is the expected value; Z (I) is the test value; P (I) is the significance level.

**Table 4:** Absolute β convergence test of provincial urban land use efficiency.

	China	Western Region	Northeast Region	Central Region	Eastern Region
Constant term	−0.0033 ***	−0.0042 ***	−0.0037 ***	−0.0033 ***	−0.0025 ***
	(−4.0278)	(−3.7843)	(−3.3122)	(−2.1145)	(−1.8923)
β	−0.0491 ***	−0.0686 ***	−0.0706 ***	−0.0723 ***	−0.0789 ***
	(−12.8323)	(−11.7823)	(−12.2812)	(−14.0067)	(−13.2867)
Model specification	Fixed effect	Fixed effect	Fixed effect	Fixed effect	Fixed effect
Ajusted R^2^	0.7838	0.8108	0.8225	0.8512	0.8826
Convergence judgment	Convergence	Convergence	Convergence	Convergence	Convergence

Note: *** indicates that the estimated coefficient is significant at the 1% level.

**Table 5:** Conditional β convergence test of provincial urban land use efficiency.

	China	Western Region	Northeast Region	Central Region	Eastern Region
Constant term	−0.0041 ***	−0.0056 ***	−0.0051 ***	−0.0046 ***	−0.0033 ***
	(−4.7729)	(−3.9923)	(−2.9762)	(−2.6626)	(−1.7745)
β	−0.0552 ***	−0.0786 ***	−0.0811 ***	−0.0852 ***	−0.1023 ***
	(−11.7261)	(−10.4487)	(−11.6072)	(−13.6745)	(−12.0043)
*Y*_1_	−0.0622 ***	−0.0652 **	−0.0726 ***	−0.0773 ***	−0.1002 ***
	(−10.2367)	(−6.6742)	(−7.8038)	(−8.6211)	(−12.7833)
*Y*_2_	−0.0603 **	−0.0611 **	−0.0708 **	−0.0794 **	−0.1013 ***
	(−12.8831)	(−8.8829)	(−9.1218)	(−9.4223)	(−11.7729)
*Y*_3_	−0.0646 ***	−0.0634 ***	−0.0772 ***	−0.0853 ***	−0.0825 **
	(−9.9236)	(−7.7412)	(−10.7083)	(−12.8734)	(−8.0234)
*Y*_4_	−0.0563 **	−0.0886 ***	−0.0662 **	−0.0683 **	−0.0744 **
	(−7.0065)	(−9.2387)	(−9.8738)	(−10.8203)	(−6.7826)
*Y*_5_	−0.0425 **	−0.0712 **	−0.0653 **	−0.0594 **	−0.0635 **
	(−6.2653)	(−8.0078)	(−7.2356)	(−6.9934)	(−7.8933)
Model specification	Fixed effect	Fixed effect	Fixed effect	Fixed effect	Fixed effect
Ajusted R^2^	0.8092	0.8365	0.8528	0.8782	0.8951
Convergence judgment	Convergence	Convergence	Convergence	Convergence	Convergence

Note: *** and ** indicate that the estimated coefficient is significant at 1% and 5% level, respectively.

## Data Availability

Not applicable.

## References

[1] Smith P. (2018). Managing the global land resource. Proc. R. Soc. B.

[2] Wang H., Qiao L., Tian C. (2020). Utilisation efficiency of construction land in China’s coastal cities based on debt level. Complexity.

[3] Lucas M.T., Chhajed D. (2004). Applications of location analysis in agriculture: A survey. J. Oper. Res. Soc..

[4] Velasco L., Rioux L. (2010). Psychosocial approach to workplace attachment: A study carried out among hospital staff. Estud. Psicol..

[5] Zhang R.T., Jiao H.F. (2015). Urban land use efficiency pattern evolution and driving mechanism in the Yangtze river economic belt. Resour. Environ. Yangtze Basin.

[6] Bittner C., Bittner C., Sofer M. (2013). Land use changes in the rural–urban Fringe: An Israeli case study. Land Use Policy.

[7] Zheng W.W., Ke X.L., Xiao B.Y., Zhou T. (2019). Optimizing land use allocation to balance ecosystem services and economic benefits —A case study in Wuhan, China. J. Environ. Manag..

[8] Huyen C.T., Phap V.M., Nga N.T. (2021). Study on performance and economic efficiency of solar power on agricultural land: A case study in Central Region, Vietnam. Int. J. Renew. Energy Res..

[9] Zhao X.F., Lou J.J., Huang X.J., Yao L., Zhao Y.T. (2017). Research progress in urban land use efficiency. Mod. Urban Stud..

[10] David M., Eligius M.T. (2000). A framework to study nearly optimal solutions of linear programming models developed for agricultural land use exploration. Ecol. Model..

[11] Liang L.T., Zhao Q.L., Chen C. (2013). Analysis on the characters of spatial disparity of urban land use efficiency and its optimization in Chin. China Land Sci..

[12] Tone K. (2001). A slacks-based measure of efficiency in data envelopment analysis. Eur. J. Oper. Res..

[13] Wu C.Y., Wei Y.D., Huang X.L., Chen B.W. (2017). Economic transition, spatial development and urban land use efficiency in the Yangtze River Delta, China. Habitat Int..

[14] Salvati L., Zambon I., Chelli F.M., Serra P. (2018). Do spatial patterns of urbanization and land consumption reflect different socioeconomic contexts in Europe?. Sci. Total Environ..

[15] Chen W., Wu Q. (2014). Economic efficiency of urban construction land and its influential factors in Yangtze river delta. Econ. Geogr..

[16] Marshall J.D. (2007). Urban land area and population growth: A new scaling relationship for metropolitan expansion. Urban Stud..

[17] Fan P.F., Feng S.Y., Su M., Xu M.J. (2018). Differential characteristics and driving factors of land use efficiency in different functional cities based on undesirable outputs. Resour. Sci..

[18] Verburg P.H., Berkel D.B., Doorn A.M., Eupen M., Heiligenberg H.A. (2010). Trajectories of land use change in Europe: A model-base exploration of rural futures. Landsc. Ecol..

[19] Louw E., Krabbene E., Amsterdam H. (2012). The spatial productivity of industrial land. Reg. Stud..

[20] Guastella G., Pareglio S., Sckokai P. (2017). A spatial econometric analysis of land use efficiency in large and small municipalities. Land Use Policy.

[21] Koroso N.H., Zevenbergen J.A., Lengoiboni M. (2020). Urban land use efficiency in Ethiopia: An assessment of urban land use sustainability in Addis Ababa. Land Use Policy.

[22] Auzins A., Geipele I., Stamure I. (2013). Measuring land-use efficiency in land management. Adv. Mater. Res..

[23] Fang C.L., Guan X.L., Lu S.S., Zhou M., Deng Y. (2013). Input-output efficiency of urban agglomerations in China: An application of Data Envelopment Analysis (DEA). Urban Stud..

[24] Tone K. (2010). An epsilon-based measure of efficiency in DEA-a third pole of technical efficiency. Eur. J. Oper. Res..

[25] Liu S.C., Lin Y.B., Ye Y.M., Xiao W. (2021). Spatial-temporal characteristics of industrial land use efficiency in provincial China based on a stochastic frontier production function approach. J. Clean. Prod..

[26] Chen W., Chen W.J., Ning S.Y., Liu E.N., Zhou X., Wang Y.N., Zhao M.J. (2019). Exploring the industrial land use efficiency of China’s resource-based cities. Cities.

[27] Wang L.J., Li H. (2014). Cultivated land use efficiency and the regional characteristics of its influencing factors in China: By using a panel data of 281 prefectural cities and the stochastic frontier production function. Geogr. Res..

[28] Hegazy I.R., Kaloop M.R. (2015). Monitoring urban growth and land use change detection with GIS and remote sensing techniques in Daqahlia governorate Egypt. Int. J. Sustain. Built Environ..

[29] Cao X.S., Liu Y.W., Li T., Liao W. (2019). Analysis of spatial pattern evolution and influencing factors of regional land use efficiency in China based on ESDA-GWR. Sci. Rep..

[30] Yu J.Q., Zhou K., Yang S.L. (2019). Land use efficiency and influencing factors of urban agglomerations in China. Land Use Policy.

[31] Zhu X.H., Li Y., Zhang P.F., Wei Y.G., Zheng X.Y., Xie L.L. (2019). Temporal–spatial characteristics of urban land use efficiency of China’s 35mega cities based on DEA: Decomposing technology and scale efficiency. Land Use Policy.

[32] Xie H.L., Chen Q.R., Lu F.C., Wu Q., Wang W. (2018). Spatial-temporal disparities, saving potential and influential factors of industrial land use efficiency: A case study in urban agglomeration in the middle reaches of the Yangtze River. Land Use Policy.

[33] He Y.F., Xie H.L., Fan Y.H., Wang W., Xie X. (2016). Forested land use efficiency in China: Spatiotemporal patterns and influencing factors from 1999 to 2010. Sustainability.

[34] Tang Y.K., Wang K., Ji X.M., Xu H., Xiao Y.Q. (2021). Assessment and Spatial-temporal evolution analysis of urban land use efficiency under green development orientation: Case of the Yangtze River Delta Urban Agglomerations. Land.

[35] Yao M., Zhang Y. (2021). Evaluation and optimization of urban land-use efficiency: A case study in Sichuan Province of China. Sustainability.

[36] Yang X.D., Wu Y.X., Dang H. (2017). Urban land use efficiency and coordination in China. Sustainability.

[37] Ge X.J., Liu X.X. (2021). Urban land use efficiency under resource-based economic transformation—A case study of Shanxi province. Land.

[38] Lau S.Y., Giridharan R., Ganesan S. (2005). Multiple and intensive land use: Case studies in Hong Kong. Habitat. Int..

[39] Barbosa J.A., Bragança L., Mateus R. (2015). Assessment of land use efficiency using BSA tools: Development of a new index. J. Urban Plan.

[40] Liang N., Zou Z.H., Wei Y. (2019). Regression models (SVR, EMD and FastICA) in forecasting water quality of the Haihe River of China. Desalin. Water Treat..

[41] Pang Y.Y., Wang X.J. (2020). Land-use efficiency in Shandong (China): Empirical analysis based on a Super-SBM model. Sustainability.

[42] Charnes A.A., Cooper W.W., Rhodes E. (1978). Measuring the efficiency of decision making units. Eur. J. Oper. Res..

[43] Pan J.W., Chen Y.Y., Zhang Y., Chen M., Fennell S., Luan B., Wang F., Meng D., Liu Y.L., Jiao L.M. (2020). Spatial-temporal dynamics of grain yield and the potential driving factors at the county level in China. J. Clean. Prod..

[44] Cartone A., Casolani N., Liberatore L., Postiglione P. (2017). Spatial analysis of grey water in Italian cereal crops production. Land Use Policy.

[45] Xie H.L., Wang W. (2015). Spatiotemporal differences and convergence of urban industrial land use efficiency for China’s major economic zones. J. Geogr. Sci..

[46] Ge K., Zou S., Chen D.L., Lu X.H., Ke S.A. (2021). Research on the spatial differences and convergence mechanism of urban land use efficiency under the background of regional integration: A case study of the Yangtze River Economic Zone, China. Land.

[47] Yang H.R., Wu Q. (2019). Land use eco-efficiency and its convergence characteristics under the constraint of carbon emissions in China. Int. J. Environ. Res. Public Health.

[48] Zhang C.Z., Su Y.Y., Yang G.Q., Chen D.L., Yang R.X. (2020). Spatial-temporal characteristics of cultivated land use efficiency in major function-oriented zones: A case study of Zhejiang province, China. Land.

[49] Zhu X.H., Zhang P.F., Wei Y.G., Li Y., Zhao H.R. (2019). Measuring the efficiency and driving factors of urban land use based on the DEA method and the PLS-SEM model-A case study of 35 large and medium-sized cities in China. Sustain. Cities Soc..

[50] Jiang H.L. (2021). Spatial-temporal differences of industrial land use efficiency and its influencing factors for China’s central region: Analyzed by SBM model. Environ. Technol. Innov..

[51] Zhuang H.W., Li H. (2011). The evaluation research of industrial land use efficiency of different regions development zone in Hunan. Econ. Geogr..

[52] Peng C., Xiao H., Liu Y., Zhang J.J. (2017). Economic structure and environmental quality and their impact on changing land use efficiency in China. Front. Earth Sci..

[53] Liu S.C., Ye Y.M., Xaio W. (2020). Spatial-temporal differentiation of urban land-use efficiency in China based on stochastic frontier analysis. China Land Sci..

[54] Yang K., Zhong T.Y., Zhang Y., Wen Q. (2020). Total factor productivity of urban land use in China. Growth Chang..

[55] Jiang X., Lu X.H., Liu Q., Chang C., Qu L.L. (2021). The effects of land transfer marketization on the urban land use efficiency: An empirical study based on 285 cities in China. Ecol. Indic..

[56] Chen W., Shen Y., Wang Y.N., Wu Q. (2018). The effect of industrial relocation on industrial land use efficiency in china: A spatial econometrics approach. J. Clean. Prod..

